# Exogenous L-Cysteine and Its Transport Through CtaP Play a Role in Biofilm Formation, Swimming Motility, and Swarming Motility of *Listeria monocytogenes*

**DOI:** 10.3390/foods14111845

**Published:** 2025-05-22

**Authors:** Mahide Muge Yilmaz Topcam, Nattanicha Prayoonwiwat, Carolina Bruschi, Kimon Andreas G. Karatzas

**Affiliations:** Department of Food and Nutritional Sciences, School of Chemistry, Food and Pharmacy, University of Reading, Reading RG6 6AD, UK; m.yilmaz@pgr.reading.ac.uk (M.M.Y.T.); nattanicha.p@dld.go.th (N.P.); c.bruschisilva@yahoo.com (C.B.)

**Keywords:** *Listeria monocytogenes*, bacterial adhesion, flagellar motility, *ctaP*, cysteine transporter, quorum sensing, environmental sensing

## Abstract

*Listeria monocytogenes* is of a significant concern for the food industry, largely due to its ability to form biofilms. Flagellar motility and environmental factors are crucial for biofilm formation. Cysteine is an important compound affecting the behavior of this bacterium; therefore, we investigated its role in growth, biofilm formation and motility of *L. monocytogenes* 10403S through a mutant in cysteine uptake (Δ*ctaP*). Basal defined media (DM) and L-cysteine-supplemented DM were used. Biofilm formation was promoted by L-cysteine supplementation in both wild type (WT) and Δ*ctaP*. Lower biofilm formation of Δ*ctaP* compared to WT indicates the significance of the cysteine transporter and cysteine uptake. A negative correlation was found between growth and biofilm formation, especially in the presence of high L-cysteine concentrations. Motility experiments showed that as the L-cysteine concentration increased, the swarming motility of WT decreased. Furthermore, swimming motility of WT was enhanced with L-cysteine supplementation, while the swimming motility of Δ*ctaP* remained unaffected. To evaluate the role of cysteine and CtaP in biofilm formation and motility, transcriptome analysis, comparing WT and Δ*ctaP* in basal and L-cysteine-supplemented (1.57 and 3.67 mM) DM, was conducted at 37 °C. The investigation of biofilm-related genes explained the role of *ctaP* and revealed induced expression of flagella and chemotaxis genes by L-cysteine.

## 1. Introduction

*Listeria monocytogenes* is a Gram-positive bacterium that can thrive in both aerobic and facultative anaerobic conditions. It is the third most harmful zoonotic pathogen that is responsible for listeriosis, affecting humans and animals through contaminated food [1,2]. *L. monocytogenes* has emerged as a frequent contaminant in foods, posing a threat primarily to consumers and the food industry. Upon ingestion, this pathogen can cross epithelial barriers, invade cells, and replicate inside them by manipulating virulence factors and spread through the body of the host, causing the serious disease listeriosis [3]. Although listeriosis outbreaks were first reported in the 1980s, most major incidents have occurred over the past two decades, with an increasing frequency worldwide [4,5,6,7]. Recent surveillance reports confirm that listeriosis remains a significant global health concern, particularly in vulnerable populations. Data from the European Food Safety Authority (EFSA) and the U.S. Centers for Disease Control and Prevention (CDC) show a steady number of confirmed cases in recent years [4,8], highlighting the significance of food safety practices and continued monitoring.

Numerous listeriosis outbreaks have been linked to milk and dairy products, meat and meat-based items, and fresh produce [2,9] that contain high concentrations of cysteine [10,11]. Its ability to withstand diverse environmental factors contributes to its presence and persistence in foods resulting in cases and outbreaks of listeriosis. This is mostly attributed to the rapid biofilm formation on various surfaces within food processing environments and some strains have developed resistance to different disinfectants [2]. Biofilms are communities of microorganisms that adhere to surfaces and undergo significant changes as they transition from a planktonic state to biofilm communities [12]. The capacity of *L. monocytogenes* to survive and persist for extended periods in food industry equipment and environments, especially under challenging conditions, is likely linked to its ability to form biofilms. Therefore, the latter is a key focus of research in food safety [13].

Flagellar motility plays a significant role in the survival of *L. monocytogenes* outside the host, which is particularly important for nutrient acquisition through chemotaxis, and it facilitates biofilm formation, contributing to bacterial persistence in the environment [14,15,16]. Bacterial motility and biofilm formation are influenced by environmental factors, including various chemical, physical, and biological stimuli. Bacteria capable of self-movement have the advantage of navigating towards nutrients, or away from harmful substances, enabling them to actively seek environments optimal for growth and form complex communities that enhance their survival [17]. Among the defined motility behaviors, swimming and swarming motility are the only ones directly related to flagella [9]. The availability and abundance of environmental nutrients influence motility, biofilm formation, and colonization [18,19,20,21]. *L. monocytogenes* strains were able to form higher biofilms in a nutrient-limited environment than in nutrient-rich environments [19,20]. While many organisms are non-motile in minimal media [18], supplementation with amino acids such as glutamate, aspartate, histidine, and proline can induce swarming motility [22].

Moreover, nutrient availability and quorum sensing (QS) work together to influence the biofilm formation of *L. monocytogenes* [23]. While there is limited research specifically addressing the role of cysteine and its metabolism in *L. monocytogenes* biofilm formation, various studies suggest potential links. *L. monocytogenes* possesses several genes encoding transporters to fulfil nutrient requirements. The Ctp complex, comprising CtaP and permeases CtpP1 and CtpP2, is one of the cysteine transporters in *L. monocytogenes* and it plays a role in growth, virulence, and attachment to host cells [24,25]. However, the role of cysteine and its transport via any channel remains unclear.

Given the scarcity of data on *L. monocytogenes*, insights from other bacterial species can provide useful information. The CymR regulon, which is responsible for the cysteine metabolism gene expression based on cysteine availability, affects environmental signals and other QS activities [26]. Loss of CymR was found to decrease biofilm formation of *Staphylococcus aureus* [27], suggesting a broader role for cysteine metabolism in biofilm development. Furthermore, cysteine biosynthesis is essential for swarming motility but not swimming motility of various bacteria, including *Salmonella typhimurium* and *Serratia marcescens* [28,29]. This requirement is associated with phospholipase activity and a decrease in the transcription of the flagellar regulator genes *flhD* and *fliA* [29]. Additionally, cysteine biosynthesis and transport of exogenous cysteine into the cell affect biofilm formation. For example, the mutation in the *cj0025c* gene encoding a cysteine transporter in *Campylobacter jejuni* leads to reduced motility and biofilm formation [30]. These findings highlight the importance of cysteine metabolism in bacterial behavior, raising the possibility that similar mechanisms may be at play in *L. monocytogenes*.

While the role of environmental factors in the biofilm formation and motility of *L. monocytogenes* has been increasingly recognized [13], the effect of cysteine remains poorly understood. Despite evidence from other bacterial species such as *Staphylococcus aureus*, *Salmonella typhimurium*, *Serratia marcescens*, and *Campylobacter jejuni* [27,28,29,30] suggesting that cysteine metabolism and transport influence biofilm and motility, there is a lack of studies directly investigating this in *L. monocytogenes*. Notably, the role of cysteine transporter CtaP on biofilm formation and flagellar motility in *L. monocytogenes* remains unexplored. Therefore, in the present study, we aimed to investigate the effect of extracellular cysteine (including different concentrations) and the role of its transporter CtaP on biofilm formation and both swimming and swarming motility of *L. monocytogenes* 10403S. To further elucidate the underlying mechanisms, we also conducted a transcriptomic analysis to understand how exogenous cysteine and its transport affect these characteristics in *L. monocytogenes*.

## 2. Materials and Methods

### 2.1. Bacterial Strains and Growth Conditions

*L. monocytogenes* 10403S WT and its isogenic Δ*ctaP* mutant were used in this study (Table 1). Stock cultures were stored at −80 °C in 7% (*v*/*v*) dimethyl sulfoxide (DMSO; Sigma-Aldrich Dorset, UK). Prior to the experiments, stock cultures were streaked onto brain heart infusion (BHI) agar (Neogen, UK) and incubated overnight at 37 °C. Three single colonies were transferred into 3 mL of BHI broth and were incubated at 37 °C overnight with shaking at 120 RPM. Five μg/mL erythromycin (Sigma-Aldrich, Dorset, UK) was added into BHI broth for Δ*ctaP*.

### 2.2. Determination of Planktonic Growth

In this work, we aimed to look at the effects of different concentrations of L-cysteine in Defined media (DM). However, *L. monocytogenes* is unable to grow without L-cysteine and, therefore, defined media (DM) prepared according to Amezaga et al., 1995 [32]. Subsequently, whenever we mention DM, we refer to the basal version containing the above concentration of cysteine (0.82 mM). In addition to the above, different versions of DM containing gradually increasing final L-cysteine concentrations of 1.57, 3.67, 6.51, and 12.21 mM were prepared. These L-cysteine concentrations were achieved by volumetric adjustment (*v*/*v*) of a L-cysteine hydrochloride monohydrate (Sigma-Aldrich, Dorset, UK) stock solution (10 μg/mL), resulting in multiple concentrations of the actual concentration present in DM (0.82 mM) to assess dose-dependent effects. *L. monocytogenes* strains were initially grown in 3 mL BHI broth (Neogen, UK) at 37 °C overnight as mentioned in Section 2.1. Subsequently, 20 µL of overnight cultures were transferred into 230 µL of DM and L-cysteine-supplemented DMs in a 96-well plate. Negative controls without *L. monocytogenes* were included for each L-cysteine concentration. After placing the lids on top of the plates, parafilm was used to seal the edges. Plates were incubated at 30 °C and 37 °C for 24 h and cell turbidity was measured using a microtiter plate reader (FLUOstar Omega, BMG Labtech, Ortenberg, Germany), at an optical density of 595 nm (OD_595_) for the determination of growth. The average OD_595_ value from the controls on each microtiter plate was subtracted from the average OD_595_ value of each test strain on the same plate for blank correction. Each assay was repeated three times for each strain and the mean OD_595_ values along with standard deviations were calculated. All experiments were performed in three biological and three technical replicates.

### 2.3. Determination of Biofilm Formation

The biofilm formed by the cultures described in Section 2.2 was used for the following step. Subsequently, after 24 h of growth, supernatants were removed by pipetting from each well. Wells were rinsed three times with 250 µL sterilized distilled water. Plates were dried in an upside-down position for 30 min. Then, staining was conducted with the use of 200 µL of 0.1% crystal violet (CV). Plates were covered with parafilm and incubated at room temperature for 45 min. The rinsing step was repeated three times with 250 µL sterilized distilled water. Two hundred and ten µL of 95% ethanol were added to each well and plates were incubated at 4 °C for 30 min for CV stabilization. Two hundred µL of each well was transferred into a sterile 96 well-plate to be measured using a microtiter plate reader (FLUOstar Omega, BMG Labtech, Ortenberg, Germany) at an optical density of 595 nm (OD_595_ nm). The average OD_595_ value from the controls on each microtiter plate was subtracted from the average OD_595_ value of each test strain on the same plate for blank correction. Each assay was repeated three times for each strain and the mean OD_595_ values along with standard deviations were calculated. All experiments were repeated thrice in terms of biological replicates, and for each biological replicate, three technical replicates were also used.

### 2.4. Cell Motility Assay: Defined Media Supplemented with Different Concentrations of L-Cysteine with Agar

To conduct motility assays, agar was added to distilled water at concentrations of 0.4% for swarming motility and 0.3% for swimming motility, then sterilized in glass bottles. After sterilization, bottles containing agar were placed in a hot water bath (42 °C) to keep it liquid until preparation of the motility plates. Defined media agar (DM) was prepared as previously described (Section 2.2) with the addition of 5 mg/mL glucose and 169 μg/mL L-glutamic acid monosodium salt hydrate (Sigma-Aldrich, Dorset, UK). Ten μg/mL L-cysteine hydrochloride monohydrate stock solution (Sigma-Aldrich, Dorset, UK) was used to prepare semi-solid agar plates and supplemented with gradually increasing cysteine concentrations (*v*/*v*) with a final concentration of 1.57 mM, 3.67 mM, 6.51 mM, and 12.21 mM.

Twenty ml of each semi-solid DM containing different L-cysteine concentrations were poured into plates and left to solidify. Overnight cultures were stabbed onto the plates. Motility was assessed under aerobic conditions for 3 days and motility diameters were measured in centimeters (cm). To evaluate the effect of temperature-dependent behavior, focusing on environmental conditions, plates were incubated at 20, 25, 30, and 37 °C. The latter temperature was included as it is also the temperature of many warm-blooded animals.

### 2.5. RNA Sequencing (RNA-Seq) Sample Preparation and Analysis of L. monocytogenes 10403S WT and ΔctaP in DMs

Overnight cultures of *L. monocytogenes* 10403S WT and Δ*ctaP* were inoculated (1% *v*/*v*) in 20 mL DM or DM supplemented with 1.57 and 3.67 mM cysteine. Cultures were incubated at 37 °C for 17 h. After growth, 16.66 mL of cultures were mixed with 3.33 mL phenol/ethanol (5/95%) solution. Cell suspensions were centrifuged at 5000× *g* for 10 min (4 °C). The supernatant was discarded and the cell pellets were frozen at –80 °C until further processing. RNA was isolated with RNeasy Mini Kit (Qiagen, Manchester, UK) and possible contamination with genomic DNA was eliminated through Turbo DNA-free™ Kit (Quiagen, Manchester, UK). RNA purity was assessed using the NanoPhotometerR spectrophotometer (IMPLEN, Westlake Village, CA, USA). All samples passed quality control (the A260/A280 Ratio is around 2.0 for pure RNA and the A260/A230 Ratio is 2.0 for contamination) and were further processed. RNA sequencing (RNA-Seq) and data analysis were performed by Novogene (Hong Kong, China).

To prepare RNA samples, 3 µg of RNA was used in total. Sequencing libraries were created using the NEBNext^®^ Ultra™ Directional RNA Library Prep Kit for Illumina^®^ (NEB, Ipswich, MA, USA), with unique index codes assigned to each sample. mRNA was isolated from the total RNA using poly-T oligo-attached magnetic beads, while rRNA was removed with a specialized kit. The RNA was fragmented using divalent cations at high temperatures in NEBNext First Strand Synthesis Reaction Buffer (5X). First-strand cDNA was synthesized using a random hexamer primer and M-MuLV Reverse Transcriptase (RNaseH), followed by second-strand cDNA synthesis using DNA Polymerase I and RNase H, with dUTP replacing dTTP. The resulting overhangs were converted into blunt ends using exonuclease and polymerase. After adding adenine to the 3′ ends, the NEBNext Adaptor with a hairpin loop structure was ligated to prepare the fragments for hybridization. cDNA fragments of about 150–200 bp were selected and purified using the AMPure XP system (Beckman Coulter, Beverly, CA, USA). Then, 3 µL of USER Enzyme (NEB, Ipswich, MA, USA) were added to the adaptor-ligated cDNA, incubated for 15 min, and then at 95 °C for 5 min. PCR amplification was carried out with Phusion High-Fidelity DNA polymerase, Universal PCR primers, and an Index Primer. The amplified library was purified using the AMPure XP system and its quality was assessed with the Agilent Bioanalyzer 2100 system.

For differential gene expression analysis, WT and Δ*ctaP* were grown in DM and DM supplemented with cysteine (1.57 mM and 3.67 mM) under anaerobic conditions to study how cysteine transport and external cysteine affect *L. monocytogenes* gene expression.

### 2.6. Statistical Analysis

All experiments were run in triplicate and results were assessed using paired Student’s T-test and Tukey on Minitab Statistical Software (Version 21.3, Minitab LLC, State College, PA, USA). A *p*-value lower than 0.05 denotes statistically significant results.

Pearson’s correlation coefficient (r) was calculated to assess the relationship between biofilm formation and growth. Correlation coefficient r ranges from −1 to 1, where the r value close to 1 indicates a strong positive correlation, close to −1 indicates a strong negative correlation, and near 0 suggests no correlation. Statistically, significance was set at *p* < 0.05.

Statistical analysis for transcriptomic analysis, including three replicates from each group, was performed using the DESeq R package (version 1.18.0), applying a negative binomial distribution model to identify differentially expressed genes. The *p*-values were adjusted using the Benjamini–Hochberg method and genes with an adjusted *p*-value below 0.05 were considered differentially expressed.

## 3. Results

### 3.1. Planktonic Growth at the End of the 24 h Period

**Figure 1:**
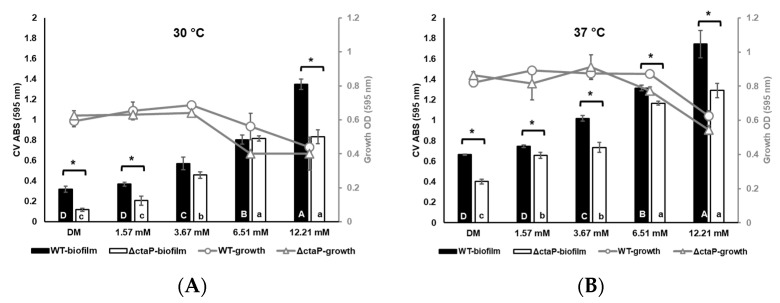
Biofilm formation (bars) and growth (lines) of WT (black bars and round markers) and Δ*ctaP* (white bars and triangle markers) in DMs supplemented with different L-cysteine concentrations were measured after 24 h of growth at 30 °C (**A**) and 37 °C (**B**). Bars and lines are calculated from three identical biological replicates and error bars represent the standard deviation. Different upper cases show a significant difference between WT strains in DMs (*p* < 0.05). Different lower cases show a significant difference between Δ*ctaP* strains in DMs (*p* < 0.05). Asterisks indicate a significant difference between WT and Δ*ctaP* strains in the same media (*p* < 0.05).

The final OD_595_ of growth was measured before CV staining. Low L-cysteine supplementation slightly promoted the growth of WT and Δ*ctaP* (*p* > 0.05; Figure 1) at the end of 24 h. On the other hand, the growth of both WT and Δ*ctaP* in the presence of higher concentrations of L-cysteine (6.51 and 12.21 mM) resulted in lower OD_595_ (*p* < 0.05; Figure 1). The difference between OD_595_ values of WT and Δ*ctaP* in DMs was not significant at both 30 and 37 °C (*p* > 0.05; Figure 1). However, both WT and Δ*ctaP* had higher OD at 37 °C than at 30 °C (*p* < 0.05; Figure 1).

### 3.2. Biofilm Formation at the End of the 24 h Period

Following the determination of growth, biofilm formation of 10403S WT and Δ*ctaP* was also investigated in different cysteine concentrations (DM; 0.82 mM and cysteine supplementation with a final concentration of 1.57, 3.67, 6.51 and 12.21 mM) at 30 and 37 °C. As the temperature increased, the biofilm formation enhanced (Figure 1A,B). Meanwhile, the biofilm formation patterns for both WT and Δ*ctaP* were found to be similar at both 30 and 37 °C.

The lowest biofilm formation of WT was observed in basal DM compared to L-cysteine-supplemented DMs (with 0.320 ABS_595_ at 30 °C and 0.664 ABS_595_ at 37 °C; *p* < 0.05; Figure 1A,B). Supplementation of DM with 1.57 mM L-cysteine did not affect the biofilm formation of WT (*p* > 0.05) in comparison to basal DM (at both temperatures). Nonetheless, the other L-cysteine concentrations (3.67 mM, 6.51 mM, and 12.21 mM) promoted biofilm formation significantly (*p* < 0.05; Figure 1A,B). Δ*ctaP* formed less biofilm than the WT in all the L-cysteine supplementation levels (*p* < 0.05; Figure 1). Similar to WT, increased L-cysteine concentrations resulted in higher biofilm formation for Δ*ctaP* (*p* < 0.05; Figure 1A,B).

### 3.3. Relationship Between Growth and Biofilm Formation of L. monocytogenes

Pearson correlation analysis was used to further understand the relationship between growth and biofilm formation in the presence of different L-cysteine concentrations. The correlation analysis of biofilm formation and growth data showed a significant negative correlation for both WT and Δ*ctaP* at 30 and 37 °C (−0.72 and −0.68 for WT and −0.87 and −0.70 for Δ*ctaP*; *p* < 0.05; Table 2). When analyzing growth conditions separately, the correlation between growth and biofilm formation of WT in basal DM and 1.57 mM L-cysteine had a positive correlation at both 30 °C and 37 °C (*p* > 0.05; Table 2). On the other hand, L-cysteine supplementation resulted in a strong negative correlation (with −0.99, *p* < 0.05; Table 2) between bacterial growth and biofilm formation in WT in the presence of 3.67 mM L-cysteine at 30 °C. The same pattern for WT was also observed at 37 °C; however, all the correlations were weak (*p* > 0.05; Table 2). The correlation between growth and biofilm formation of Δ*ctaP* in basal DM was found to be positive but not statistically significant (*p* > 0.05; Table 2). L-cysteine supplementation, regardless of the concentration, resulted in a negative correlation between growth and biofilm formation for Δ*ctaP* at 30 °C (*p* > 0.05; Table 2). However, a strong negative correlation between growth and biofilm formation was observed specifically in 12.21 mM L-cysteine (*p* < 0.05; Table 2). Controversially, at 37 °C, 12.21 mM L-cysteine resulted in a strong positive correlation for Δ*ctaP* with 0.99 (*p* < 0.05; Table 2).

### 3.4. Effect of Cysteine on Swarming and Swimming Motility of L. monocytogenes 10403S WT and ΔctaP

**Figure 2:**
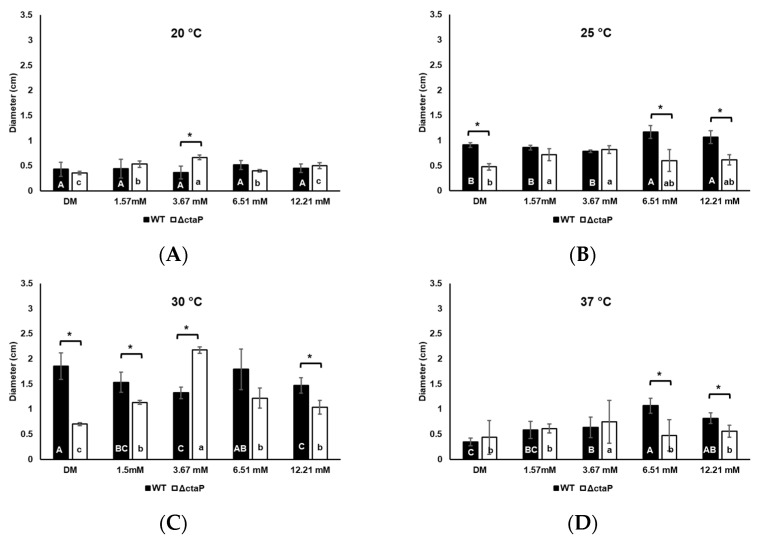
Swarming motility of WT (black bars) and Δ*ctaP* (white bars) cells in DM plates supplemented with different L-cysteine concentrations after 3 days of incubation at 20 °C (**A**), 25 °C (**B**), 30 °C (**C**), and 37 °C (**D**). Bars calculated from three biological replicates and error bars represent the standard deviation. Different upper cases show a significant difference between WT strains in DMs (*p* < 0.05). Different lower cases show a significant difference between Δ*ctaP* strains in DMs (*p* < 0.05). Asterisks indicate a significant difference between WT and Δ*ctaP* strains in the same media (*p* < 0.05).

Swarming and swimming motility of *L. monocytogenes* 10403S were investigated in DM supplemented with gradually increasing concentrations of L-cysteine. To investigate the role of temperature on swimming and swarming motility, we incubated cells at 20, 25, 30, and 37 °C. The highest swarming motility was observed at 30 °C for WT, followed by 25, 20, and 37 °C, respectively (Figure 2). The motility of WT was relatively higher at 37 °C compared to 20 °C (*p* > 0.05; Figure 2A,D).

With the addition of low-level L-cysteine (1.57 and 3.67 mM) in the environment, the swarming motility of *L. monocytogenes* 10403S WT decreased significantly at 30 °C (*p* < 0.05; Figure 2C). High levels of L-cysteine (6.51 and 12.21 mM) led to higher swarming motility compared to 1.57 and 3.67 mM of L-cysteine supplementation (*p* > 0.05; Figure 2C). Yet, swarming motility zones on those high levels of L-cysteine were still smaller than in basal DM (*p* < 0.05; Figure 2C). The same behavior was observed at 25 °C (Figure 2B). While L-cysteine supplementation did not significantly influence the swarming motility of WT at 20 °C, increased motility was observed at 37 °C (Figure 2D).

**Figure 3:**
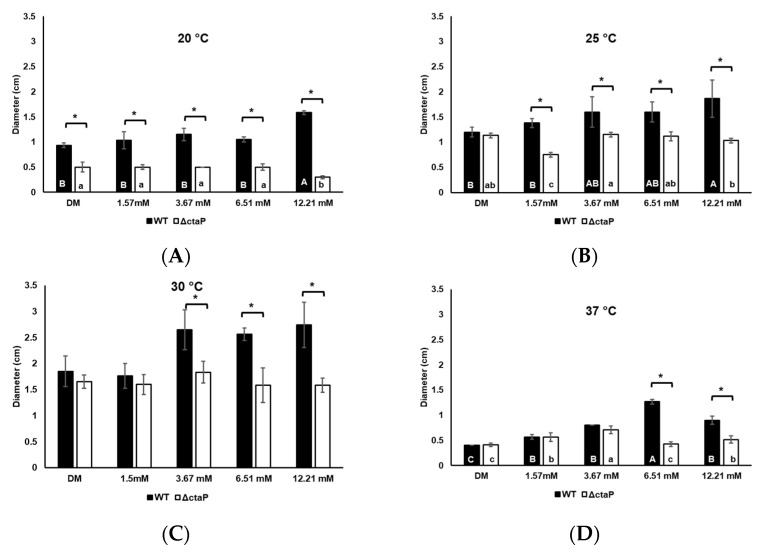
Swimming motility of WT (black bars) and Δ*ctaP* (white bars) cells in DM plates supplemented with different L-cysteine concentrations after 3 days of incubation at 20 °C (**A**), 25 °C (**B**), 30 °C (**C**), and 37 °C (**D**). Bars calculated from three biological replicates and error bars represent standard deviation. Different upper cases show a significant difference between WT strains in DMs (*p* < 0.05). Different lower cases show a significant difference between Δ*ctaP* strains in DMs (*p* < 0.05). Asterisks indicate a significant difference between WT and Δ*ctaP* strains in the same media (*p* < 0.05).

We also investigated the effect of cysteine on swimming motility. L-cysteine supplementation did not affect the swimming motility behavior of WT at 20 °C (*p* > 0.05; Figure 3A). Also, supplementation with 1.57 mM L-cysteine did not affect the swimming motility of WT compared to that of basal DM at 25 °C and 30 °C. At those temperatures, concentrations of L-cysteine higher than 1.57 mM increased the swimming motility significantly (*p* < 0.05; Figure 3B,C). Similarly to swarming motility, a slightly increased swimming motility was observed with L-cysteine supplementation at 37 °C (Figure 3D).

The 10403S Δ*ctaP* was investigated to find out the role of cysteine intake in motility. Δ*ctaP* showed significantly decreased motility compared to WT on both swimming and swarming plates. With the addition of L-cysteine in DM, 10403S WT showed proportionally higher swarming motility than Δ*ctaP* (*p* < 0.05; Figure 2). The 10403S Δ*ctaP* showed the highest swarming motility on DM agar with a final concentration of 3.67 mM L-cysteine, with a 2.2 cm at 30 °C (*p* < 0.05; Figure 2C). Although higher levels of L-cysteine (6.51 mM and 12.21 mM) reduced swarming motility compared to plates with 3.67 mM L-cysteine, the ΔctaP exhibited increased swarming motility with additional L-cysteine at 30 °C (*p* < 0.05; Figure 2C). On the other hand, L-cysteine supplementation, regardless of the concentration, did not affect the swimming motility of Δ*ctaP* at 20 °C and 30 °C (*p* > 0.05; Figure 3A,B). Although the swimming motility of Δ*ctaP* at 25 °C showed a significant drop in DM with 1.57 mM L-cysteine (*p* < 0.05; Figure 3B), similar swimming motility was observed on other L-cysteine concentrations tested (*p* > 0.05; Figure 3B). L-cysteine supplementation increased swimming motility of Δ*ctaP* at 37 °C (*p* < 0.05; Figure 3D); however, 6.51 mM L-cysteine had no effect (*p* > 0.05; Figure 3D).

### 3.5. Transcriptomic Analysis

Transcriptomic analyses comparing (i) *L. monocytogenes* 10403S WT vs. Δ*ctaP* in basal DM, (ii) WT grown in basal DM vs. DM with 1.57 mM L-cysteine and (iii) WT grown in basal DM vs. 3.67 mM L-cysteine were conducted. The summary of the transcriptome assembly statistics is shown in the supplementary data (Table S1). The error rate of a single base location sequencing was less than 1% in all groups. The Q2 and Q3 were equal to or higher than 97% or 93%, respectively. Supplementation with 1.57 mM L-cysteine resulted in the upregulation of 1485 and downregulation of 1450 genes in WT compared to basal DM (Figure S1). Moreover, with the 3.67 mM L-cysteine supplementation, 1122 genes were upregulated and 1165 genes were downregulated in WT compared to basal DM (Figure S1). The deletion of *ctaP* resulted in the upregulation of 835 genes and the downregulation of 602 genes in basal DM (Figure S1). Genes involved in biofilm formation, flagellar assembly (KEGG; lmt02040), and bacterial chemotaxis (KEGG; lmt02030) were investigated to assess their roles in biofilm formation and motility.

Genes previously associated with biofilm formation in *L. monocytogenes* [33,34] were investigated (Table S2). *hly* (encoding virulence factor listeriolysin O [35]), *bdlA* (responsible for biofilm detachment), *dltA*, *dltB* (encoding D-alanylation of extracellular lipoteichoic acids), and *phoR* (regulatory gene) were some of the genes that showed significantly altered expression in all compared conditions (*padj* < 0.05; Table 3). In addition, the expression of the CymR regulon, TcyKLMN transporter complex, and *luxS* (S-Ribosylhomocysteinase) was also investigated due to their role in cysteine metabolism.

*(i)*  Comparison of Δ*ctaP* vs. WT in Basal DM

In Δ*ctaP*, several biofilm-related genes exhibited significant expression changes compared to WT (*padj* < 0.05; Table 3). While *hly* was significantly downregulated (*padj* < 0.05; Table 3), *cymR* and its regulon (TcyKLMN complex genes) were significantly upregulated (*padj* < 0.05; Table 4). The opp operon (*oppBCD*), responsible for oligopeptide transport, was significantly downregulated in Δ*ctaP* compared to WT in basal DM (*padj* < 0.05; Table 4). Moreover, the loss of *ctaP* did not affect the transcription of *luxS* in basal DM (*padj* > 0.05; Table 4).

In terms of chemotaxis, *cheR* (Table S3), *motA*, and *motB* were upregulated (*padj* < 0.05; Table 5). Interestingly, Δ*ctaP* did not show significant changes in the transcription of flagellar assembly genes compared to WT in basal DM.

*(ii)*  Comparison of WT in Basal DM vs. DM with 1.57 mM L-cysteine

L-cysteine supplementation (1.57 mM) led to significant changes in biofilm formation and motility-related genes. The key biofilm-related gene *bdlA* (involved in biofilm detachment) and the virulence-associated gene *hly* were downregulated (Table 3). *dltA*, *dltB* (involved in the D-alanylation of extracellular lipoteichoic acids) and the regulatory gene *phoR* were also downregulated (*padj* < 0.05; Table 3). The CymR regulon was downregulated (*padj* < 0.05; Table 4). The *luxS* gene was also significantly downregulated (*padj* < 0.05; Table 4).

From the flagellar assembly pathway, *motA*, *motB* (Table 5), *flgF*, and *flhA* were significantly upregulated, while *flgB*, *fliS*, *rpoD*, *flgE,* and *flgL* were significantly downregulated (*padj* < 0.05; Table S3). Moreover, *cheA* and genes encoding flagella motor switch proteins (Table 5) were significantly downregulated (*padj* < 0.05).

*(iii)*  Comparison of WT in Basal DM vs. DM with 3.67 mM L-cysteine

The CymR regulon and the TcyKLMN operon remained downregulated in WT with 3.67 mM L-cysteine supplementation compared to basal DM (*padj* < 0.05; Table 4). The *opp* operon showed further repression (*padj* < 0.05; Table 4). All the flagellar assembly genes mentioned above were downregulated in WT with the 3.67 mM L-cysteine supplementation compared to basal DM (*padj* < 0.05; Table S3). Moreover, all bacterial chemotaxis genes were significantly downregulated (*padj* < 0.05; some of them presented in Table S3).

## 4. Discussion

It is known that nutrient availability affects the biofilm formation of microorganisms, including *L. monocytogenes*. Although research directly examining the role of cysteine and its metabolism in *L. monocytogenes* biofilm formation is limited, several studies indicate possible connections. For instance, Xayarath et al., 2009, have demonstrated the involvement of the CtaP cysteine transporter in the adhesion of *L. monocytogenes* to the host cells [24]. Also, the effect of cysteine biosynthesis on biofilm formation [27,30] and the swimming and swarming motility [28,29,36] of various bacteria has been reported previously. However, the specific impact of cysteine and its transport on biofilm formation and motility has never been studied in *L. monocytogenes* before. Therefore, in this study, the role of extracellular cysteine and its transport on biofilm formation, swarming and swimming motility of *L. monocytogenes* was investigated.

The significantly lower biofilm formation of Δ*ctaP* compared to WT in all media (Figure 1A,B) clearly indicates that *ctaP* plays a critical role in the biofilm formation of *L. monocytogenes*, regardless of temperature. Our data indicate that while L-cysteine supplementation did not significantly affect the growth at lower concentrations (1.57 mM and 3.67 mM), higher concentrations (6.51 mM and 12.21 mM) led to reduced growth but significantly enhanced biofilm formation at both 30 °C and 37 °C (*p* < 0.05; Figure 1). Notably, biofilm formation increased as growth decreased, resulting in a negative correlation. These negative correlations between biofilm formation and growth have also been confirmed in *L. monocytogenes* previously [37], particularly in a cysteine-rich environment. One possible explanation is that high concentrations of L-cysteine may induce cell stress or toxicity [38], leading to increased cell death and the release of intracellular components, including extracellular DNA (eDNA) [39,40] which can facilitate adhesion and biofilm stability and formation [41,42], despite the reduced bacterial growth [43]. Although the dose-dependent effect of L-cysteine on eDNA in fermenters has been shown [44], we did not measure eDNA in the present study and we plan to assess this in further investigations aiming to identify the mechanism(s) underlying the enhanced biofilm formation observed at higher cysteine concentrations. However, as a first approach in the present study, we conducted a transcriptomic analysis at lower concentrations.

Biofilm formation is important for the survival and resilience of *L. monocytogenes* in the environment. However, environmental temperatures can be relatively high during the summer, while they are consistently high in tropical regions, where 40% of the human population of the Earth resides [45]. Higher temperatures have been shown to induce stronger biofilm formation, with biofilms acting as a protective barrier that provides a stable habitat for survival and metabolism. Raw materials containing biofilms enter production lines and could be a source of contamination and lead to the formation of further biofilms in production facilities [45,46,47,48]. Recent increases in listeriosis cases have been linked to cross-contamination in food facilities, with biofilm formation playing a key role [49]. Park et al., 2022, found that biofilm formation of *L. monocytogenes* varies among strains, with some showing strong biofilm production at 37 °C, which may enhance adhesion to epithelial cells and contribute to human infections [50]. Our findings show that the highest biofilm formation for both WT and Δ*ctaP* was observed at 37 °C and L-cysteine supplementation enhanced biofilm formation of those strains. These results align with previous studies reporting optimal biofilm formation at this temperature [50,51,52]. Therefore, we conducted a transcriptomic analysis at 37 °C comparing WT and Δ*ctaP* in the presence of different L-cysteine concentrations to examine the differential gene expression patterns and explore the relationship between biofilm formation, providing insights into the role of CtaP under varying cysteine levels.

Our transcriptomic data showed that *bdlA*, a gene crucial for biofilm detachment, was downregulated significantly in the WT with 1.57 and 3.67 mM L-cysteine supplementation (*padj* < 0.05; Table 3). Currently, no studies have explored the relationship between *bdlA* and intracellular c-di-GMP concentrations (cyclic di-guanylate monophosphate, a second messenger known to regulate biofilm formation and motility [53]) in *L. monocytogenes*. However, in the Gram-negative bacterium *Pseudomonas aeruginosa*, loss of *bdlA* has been associated with increased adhesion and elevated c-di-GMP levels [54]. The c-di-GMP regulatory pathways play a crucial role in determining whether bacteria will adopt a motile or sessile lifestyle [53] by activating the biosynthesis of exopolysaccharides [55]. Higher levels of c-di-GMP increase the adhesion of cells [56,57]. Therefore, the downregulation of *bdlA* (*padj* < 0.05; Table 3) could potentially lead to higher c-di-GMP levels [54] and enhanced attachment, resulting in higher biofilm formation. While this has been demonstrated in *Pseudomonas aeruginosa* [54], further investigation is needed to confirm whether a similar mechanism occurs in *L. monocytogenes*.

Biofilm formation stimulator VEG gene (*lmo0189*) was significantly upregulated with 3.67 mM L-cysteine (*padj* < 0.05; Table 3) but was not expressed significantly in the presence of 1.57 mM L-cysteine (*padj* > 0.05). *hly*, encoding the main virulence factor listeriolysin O (LLO), plays a role in biofilm formation [35]. Similarly, *hly* was significantly upregulated in the presence of 3.67 mM L-cysteine (*padj* < 0.05; Table 3), supporting previous findings on the promoting effects of cysteine on *hly* expression and general virulence of *L. monocytogenes* [58,59,60]. These data provide further insight into the mechanisms underlying the enhanced biofilm formation observed in WT strains grown in media supplemented with 1.57 mM and 3.67 mM L-cysteine.

Our data show that both *dltA* and *dltB* were significantly downregulated in Δ*ctaP* (*padj* < 0.05; Table 3). These genes are required for the D-alanylation of extracellular lipoteichoic acids, which is essential for maintaining proper surface charge, attachment and biofilm formation [34]. In addition to this, L-cysteine supplementation of Δ*ctaP* did not affect the transcription of these genes (Table S5). All the above suggest that *ctaP* plays a role in the expression of *dltA*/*dltB*, through an unknown regulatory mechanism. Overall, the downregulation of *dltA*, *dltB*, and regulatory gene *phoR* (responsible for the biofilm formation of *L. monocytogenes* [34]) could explain the lower biofilm formation of Δ*ctaP* compared to WT. This aligns with previous findings indicating that CtaP influences the adherence of *L. monocytogenes* to host cells [24,25]. Additionally, recent studies indicate that *ctaP* expression is specifically induced in sessile *L. monocytogenes* cells but not in planktonic cells [23], further supporting its role in biofilm formation.

Cysteine supplementation restored the biofilm formation of Δ*ctaP*, although the biofilm levels remained lower than those observed in WT (Figure 1B). This is likely due to the cysteine uptake by alternative transporters, such as the TcyKLMN complex, which is controlled by CymR [60], in response to cysteine levels [26]. CymR regulon was upregulated significantly (*padj* < 0.05; Table 4) in Δ*ctaP* in basal DM, but L-cysteine supplementation led to its downregulation (*padj* < 0.05; Table S4). Accordingly, loss of *ctaP* upregulated all *tcy* genes significantly in basal DM (*padj* < 0.05; Table 4), highlighting the compensatory role of the TcyKLMN complex under cysteine-limiting conditions [60]. Our transcriptomic data also revealed that cysteine supplementation of Δ*ctaP* downregulated the opp operon (*padj* < 0.05; Table S4), which is responsible for the transport of cysteine-containing peptides [58,61]. The exact role of TcyKLMN complex genes in the presence of various cysteine concentrations still requires further investigation. However, the expression of the CymR regulon indicates that cysteine is transported through other channels in Δ*ctaP* cells, resulting in increased biofilm formation. Our findings suggest that the CymR regulon is responsive to high cysteine availability while work in *S. aureus* has shown that it plays a role in biofilm formation [27]. It remains to be investigated if CymR plays a role in biofilm formation in *L. monocytogenes,* possibly through the regulation of genes such as *dltA*/*B.*

Additionally, the downregulation of *luxS*, which contributes to cysteine biosynthesis by recycling S-adenosylmethionine (SAM) to homocysteine [26], due to L-cysteine supplementation in both WT and Δ*ctaP* (*padj* < 0.05; Table 4), might lead to decreased cysteine biosynthesis. Since a mutation in *luxS* has been shown to enhance the biofilm formation of *L. monocytogenes* [62,63,64], the downregulation of *luxS* with cysteine supplementation might contribute to increased biofilm formation. CymR regulon interacts with the PlcRa system to further influence signaling molecule production and QS activities. LuxS is responsible for synthesizing QS molecules, AI-2 [26]. Therefore, the presence of exogenous cysteine may impact this process by altering the cysteine biosynthesis pathway, as it provides an external source of cysteine, potentially reducing the requirement for the recycling of SAM through *luxS* [62,63,64]. Yet, *luxS* is not the main determinant in biofilm formation, although it contributes to it [65,66,67].

Flagellar motility, both swimming and swarming [37], is crucial for the survival of *L. monocytogenes* outside the host, contributing to nutrient acquisition through chemotaxis and biofilm formation. These characteristics enhance the bacterium’s persistence in the environment [14,15,16]. Bacterial surface motility, driven by flagella, plays a key role in the effectiveness of initial colonization and early invasion of mammalian host cells [16]. Therefore, the swimming and swarming motility of *L. monocytogenes* strains were investigated in the presence of different L-cysteine concentrations.

In *L. monocytogenes*, flagellar motility plays a significant role in the colonization on both internal and external surfaces and this capability is influenced by temperature [14,16,51]. The motility behavior of *L. monocytogenes* 10403S WT and Δ*ctaP* on DM agar plates were tested. Swimming and swarming motility of *L. monocytogenes* showed similar behavior at different temperatures (20 °C, 25 °C, and 30 °C), with the highest motility observed at 30 °C. Notably, motility at 37 °C was still comparable to that at 20 °C, consistent with previous studies [65,68,69]. At ambient temperatures (22–28 °C), *L. monocytogenes* possesses flagella and exhibits motility; however, at the physiological temperature of mammals (37 °C), it loses its flagella and therefore its motility to avoid detection by the immune system [70,71]. However, Gao et al., 2024 [65], have found that motility-related genes *motA* and *motB* showed significantly high expression at 37 °C in different species of *L. monocytogenes*. Moreover, *L. monocytogenes* isolates from food and clinical sources were able to swim and swarm at 37 °C. At the same time, they were also able to form higher amounts of biofilm [65]. Our data revealed that *motA* and *motB* significantly upregulated (*padj* < 0.05; Table 5) in Δ*ctaP* compared to WT in basal DM. Moreover, L-cysteine supplementation of WT resulted in the upregulation of those genes significantly (*padj* < 0.05; Table 5). This aligns with previous findings, where *L. monocytogenes* species capable of swimming and swarming at 37 °C also exhibited high *motA* and *motB* expression and biofilm formation [65].

The swarming motility of WT was reduced with L-cysteine supplementation, while Δ*ctaP* showed enhanced swarming motility under similar conditions (Figure 2B,C). Although transcriptomic data did not show significant changes in flagella assembly gene transcription in Δ*ctaP* compared to WT in basal DM, the upregulation of *flhA* and *flhB* in WT with L-cysteine supplementation (*padj* < 0.05; Table 5) points to a concentration-dependent effect of L-cysteine on flagellin-related genes. *flhB* is known to be essential for the transition from hook assembly to flagellin [72]. The downregulation of *flhB* in WT with 3.67 mM L-cysteine may explain the observed reduction in swarming motility. These data suggest that L-cysteine affects the motility of WT in a concentration-dependent manner by interfering with flagellin assembly. On the other side, Δ*ctaP* appears to bypass this effect, possibly due to the involvement of other cysteine transporters.

Chemotaxis genes *cheA* and *cheY* in Δ*ctaP* (vs. WT) remained unchanged in basal DM. Moreover, L-cysteine supplementation of Δ*ctaP* did not affect the expression of these genes significantly (Table S5). These data might also help to explain why the swimming motility of Δ*ctaP* remained unchanged. In contrast, these genes were significantly downregulated in WT with the L-cysteine supplementation, including both concentrations (*padj* < 0.05; Table 5), in compliance with the enhanced swimming motility of WT. Chemotaxis allows bacteria to navigate towards attractants or away from repellents by adjusting flagellar rotation. When encountering an attractant, bacteria reduce CheA activity, leading to decreased phosphorylation of CheB and CheY proteins, promoting smooth swimming and more directed movement towards the attractant [73,74], which is consistent with the enhanced motility observed in WT following cysteine supplementation.

It is worth mentioning that the motility zones observed at 37 °C could be a growth wave [75] that is triggered by the optimum growth temperature and more nutrient availability with L-cysteine addition. Even though flagellin production is minimal at 37 °C, there is also a possibility that its expression is influenced by factors beyond temperature [76]. Since osmolarity is known to play a key role in flagellin regulation [77], L-cysteine might affect motility through osmotic shifts or other regulatory pathways. In line with this, the concentration-dependent regulation of flagella assembly and chemotaxis genes, including *flhA*, *flhB*, *cheY*, and *cheA*, was observed in our study (Table 5).

Our transcriptomic data show that in *L. monocytogenes*, cysteine uptake through *ctaP* and exogenous L-cysteine (concentration-dependent; Table 5) affect phospholipase activity. *plcA* and *plcB* were downregulated in Δ*ctaP* compared to WT. The downregulation of *plcA* in Δ*ctaP* might contribute to the decrease in motility of the mutant strain. Interestingly, *plcA* was upregulated in WT in response to 3.57 mM L-cysteine supplementation (Table 5). However, according to the literature, upregulation of *plcA* in WT is expected to result in an increased swarming motility (at 30 °C) [29]. This contradicts our swarming motility findings (Figure 2C) but aligns with swimming motility (Figure 3C). This highlights a potentially complex relationship between *plcA*-mediated phospholipase activity and different motility modes. Although we also evaluated phospholipase activity on ALOA agar, the slight reduction observed in Δ*ctaP* compared to WT was not statistically significant (Figure S2). Further work, such as overexpression studies or direct activity assays, will be important to clarify the functional consequences of *plcA* regulation on the motility behaviors of *L. monocytogenes*.

## 5. Conclusions

This study highlights the effect of cysteine and its transport on biofilm formation and motility of *L. monocytogenes*. Cysteine transporter CtaP, which is required for the growth and virulence of *L. monocytogenes*, is also necessary for biofilm formation, swimming, and swarming motility in a concentration-dependent manner. An enhanced biofilm formation was observed for both WT and Δ*ctaP* under increased L-cysteine concentrations. The transcriptomic data support the increased biofilm formation in WT, while the presence of alternative cysteine or peptide transporters may contribute to the enhanced biofilm formation observed in Δ*ctaP*. The different L-cysteine concentrations affected the swimming and swarming motility of *L. monocytogenes* in opposite ways, underscoring the complexity of its regulatory role in bacterial movement. Moreover, we show that at 37 °C, where *L. monocytogenes* loses flagella, L-cysteine supplementation influences the expression of flagella assembly and chemotaxis genes in a concentration-dependent manner. In cysteine-rich environments, increased biofilm formation and motility may provide a survival advantage by enhancing surface colonization, nutrient acquisition, and resistance to environmental stressors. This adaptability is critical for *L. monocytogenes* persistence in food production settings and within the host. These findings contribute valuable insights for future research on bacterial survival, proliferation, and virulence in both industrial and host environments.

## Figures and Tables

**Table 1:** *L. monocytogenes* strains used in this work.

Strain	Relevant Properties	Reference Source
10403S	Serotype ½ a, wild type	[31]
10403S Δ*lmo0135*	10403S with Δ*lmo0135::erm* (*ΔctaP::erm*) deletion	[24]

**Table 2:** The relationship between growth and biofilm formation of *L. monocytogenes* 10403S WT and Δ*ctaP* at 30 °C and 37 °C.

	Temperature (30 °C)		Temperature (37 °C)	
**Condition**	WT Correlation (*r*)	Δ*ctaP* Correlation (*r*)	WT Correlation (*r*)	Δ*ctaP* Correlation (*r*)
**Overall**	−0.72698 *	−0.86986 *	−0.68798 *	−0.70016 *
DM	0.182	0.87	0.857	−0.207
1.57 mM	0.424	−0.721	0.96	−0.519
3.67 mM	−0.998 *	−0.84	−0.703	0.591
6.51 mM	−0.41	−0.989	0.942	0.521
12.21 mM	0.714	−0.998 *	0.784	0.997 *

* Asterisks indicate a significant difference (*padj* < 0.05).

**Table 3:** Transcription of biofilm formation-related genes in WT grown in 1.57 mM L-cysteine-containing DM and 3.67 mM L-cysteine-containing DM compared to non-supplemented DM and in Δ*ctaP* compared to WT grown in DM under anaerobic conditions.

Gene Symbol	Locus Tag	Gene ID for 10403S	Log_2_ Fold-Change WT in 1.57 mM vs. DM	Log_2_ Fold-Change WT in 3.67 mM vs. DM	Log_2_ Fold-Change Δ*ctaP* vs. WT in DM	Gene Description
*bdlA*	*lmo1699*	LMRG_RS08590	−3.11 *	−1.48 *	−1.17	Methyl-accepting chemotaxis protein
*dltA*		LMRG_RS04905	−2.19 *	+0.62 *	−1.50 *	D-alanine--poly (phosphoribitol) ligase subunit DltA
*dltB*	*lmo0973*	LMRG_RS04900	−2.30 *	−0.03	−1.78 *	D-alanyl-lipoteichoic acid biosynthesis protein DltB
*NI*	*NI*	Novel	+0.11	+4.21 *	+0.56	Biofilm formation stimulator VEG
	*lmo0189*	LMRG_RS00910	−0.56	+4.15 *	+0.63	Veg family protein
*hly*	*lmo0202*	LMRG_RS00975	−0.45	+7.38 *	−1.34 *	Cholesterol-dependent cytolysin listeriolysin O
*phoR*	*lmo2500*	LMRG_RS12690	−0.81*	−0.56 *	−0.73 *	Alkaline phosphatase synthesis sensor protein PhoR

* Asterisks indicate a significant difference (*padj* < 0.05). “−“ indicates downregulation while “+” indicates upregulation. NI: Not identified.

**Table 4:** Transcription of the genes related to cysteine transport and quorum sensing in Δ*ctaP* compared to WT in DM and WT in 1.57 mM L-cysteine-containing DM and 3.67 mM L-cysteine-containing DM compared to non-supplemented DM under anaerobic conditions.

Gene Symbol	Locus Tag	Gene ID for 10403S	Log_2_ Fold-Change WT in 1.57 mM vs. DM	Log_2_ Fold-Change WT in 3.67 mM vs. DM	Log_2_ Fold-Change Δ*ctaP* vs. WT in DM	Gene Description
*tcyK*	*lmo2349*	LMRG_RS11840	+2.02 *	−1.88 *	+2.55 *	Amino acid ABC transporter substrate-binding protein
*tcyL*	*lmo2348*	LMRG_RS11835	+2.71 *	−2.23 *	+1.54 *	Amino acid ABC transporter permease
*tcyM*	*lmo2347*	LMRG_RS11830	+1.69 *	−6.05	+1.46 *	Amino acid ABC transporter permease
*tcyN*	*lmo2346*	LMRG_RS11825	+2.02 *	−2.87 *	+1.60 *	Amino acid ABC transporter ATP-binding protein
*CymR*	*lmo1515*	LMRG_RS07540	−0.77 *	+0.86 *	+0.58 *	Rrf2 family transcriptional regulator
*oppA*	*lmo0152*	LMRG_RS00730	−3.69 *	−2.42 *	+3.15	Peptide ABC transporter substrate-binding protein PF00496: bacterial extracellular solute-binding proteins, family 5 middle
*oppB*	*lmo2195*	LMRG_RS11135	−3.01 *	−1.82 *	−0.74 *	ABC transporter permease
*oppC*		LMRG_RS11130	−2.23 *	−1.68 *	−0.45	ABC transporter permease
*oppD*		LMRG_RS11125	−2.85 *	−0.94 *	−0.08	ABC transporter ATP-binding protein
*oppF*	*lmo2192*	LMRG_RS11120	−2.64 *	−1.21 *	+0.04	ATP-binding cassette domain-containing protein
*luxS*	*lmo1288*	LMRG_RS06405	−3.45 *	−0.47 *	+0.08	S-ribosylhomocysteine lyase

* Asterisks indicate a significant difference (*padj* < 0.05). “−” indicates downregulation while “+” indicates upregulation.

**Table 5:** Transcription of flagella assembly, chemotaxis, and phospholipase genes in WT grown in 1.57 mM L-cysteine-containing DM and 3.67 mM L-cysteine-containing DM compared to non-supplemented DM and in Δ*ctaP* compared to WT in DM under anaerobic conditions.

Gene Symbol	Locus Tag	Gene ID for 10403S	Log_2_ Fold-Change WT in 1.57 mM vs. DM	Log_2_ Fold-Change WT in 3.67 mM vs. DM	Log_2_ Fold-Change Δ*ctaP* vs. WT in DM	Gene Description
*flhA*	*lmo0680*	LMRG_RS03415	+2.34 *	+1.10 *	+0.77	Flagellar biosynthesis protein FlhA
*flhB*	*lmo0679*	LMRG_RS03410	+1.26 *	−2.99 *	+0.04	Flagellar biosynthesis protein FlhB
*cheA*	*lmo0692*	LMRG_RS03475	−3.51 *	−0.72 *	−1.51	Chemotaxis protein CheA
*cheY*	*lmo0691*	LMRG_RS03470	−2.89 *	−0.36	−1.35	Chemotaxis protein CheY
*motA*	*lmo0685*	LMRG_RS03440	+2.75 *	−1.89 *	+1.27	Flagellar motor stator protein MotA
*motB*	*lmo0686*	LMRG_RS03445	+3.39 *	−1.60 *	+1.47	Flagellar motor protein MotB
*plcA*	*lmo0201*	LMRG_RS00970	−1.42 *	+5.40 *	−1.15 *	Phosphatidylinositol-specific phospholipase C
*plcB*	*lmo0205*	LMRG_RS00990	−0.18	+4.65 *	−0.74	Phosphatidylcholine phospholipase C

* Asterisks indicate a significant difference (*padj* < 0.05). “−” indicates downregulation while “+” indicates upregulation.

## Data Availability

The data generated and analyzed during this study are available from the main author (M.M.Y.T.) or the corresponding author (K.A.K.) upon reasonable request.

## Supplementary Materials

The following supporting information can be downloaded at: https://www.mdpi.com/article/10.3390/foods14111845/s1, Figure S1: Volcano plots for (**A**) *L. monocytogenes* 10403S WT vs. Δ*ctaP* in basal DM, (**B**) WT grown in basal DM vs. DM with 1.57 mM L-cysteine, and (**C**) WT grown in basal DM vs. 3.67 mM L-cysteine under anaerobic conditions; Figure S2: Phospholipase activity of *L. monocytogenes* 10403S WT and Δ*ctaP* on ALOA plates (Trafalgar Scientific, UK), incubated at 37 °C. Zones from three biological replicates were measured, and average diameters and standard deviations are presented; Table S1: Summary of RNA-seq alignment; Table S2: Transcription of biofilm formation-related genes in WT grown in 1.57 mM L-cysteine-containing DM and 3.67 mM L-cysteine-containing DM compared to non-supplemented DM, and in Δ*ctaP* compared to WT grown in DM under anaerobic conditions; Table S3: Transcription of flagella and chemotaxis genes in WT grown in 1.57 mM L-cysteine-containing DM and 3.67 mM L-cysteine-containing DM compared to non-supplemented DM, and in Δ*ctaP* compared to WT grown in DM under anaerobic conditions; Table S4: Transcription of the genes related to cysteine transport and quorum sensing in Δ*ctaP* compared to WT in DM, in 1.57 mM L-cysteine-containing DM and 3.67 mM L-cysteine-containing DM compared to non-supplemented DM under anaerobic conditions; Table S5: Transcription of *dltA*, *dltB*, *cheA* and *cheY* in Δ*ctaP* compared to WT in DM, in 1.57 mM L-cysteine-containing DM and 3.67 mM L-cysteine-containing DM compared to non-supplemented DM under anaerobic conditions

## Abbreviations

The following abbreviations are used in this manuscript:

BHI	Brain heart infusion
CV	Crystal violet
c-di-GMP	Cyclic di-guanylate monophosphate
DMSO	Dimethyl sulfoxide
DM	Defined media
eDNA	Extracellular DNA
SAM	S-adenosylmethionine
QS	Quorum sensing
