# Supplementary material for: Exogenous L-Cysteine and Its Transport Through CtaP Play a Role in Biofilm Formation, Swimming Motility, and Swarming Motility of Listeria monocytogenes
Source: Foods. 2025 May 22;14(11):1845. doi: 10.3390/foods14111845 (PMC12155340; doi:10.3390/foods14111845)
Supplement: Supplementary file 1 [file foods-14-01845-s001.zip › foods-3600443-supplementary.pdf]

Supplementary Materials:

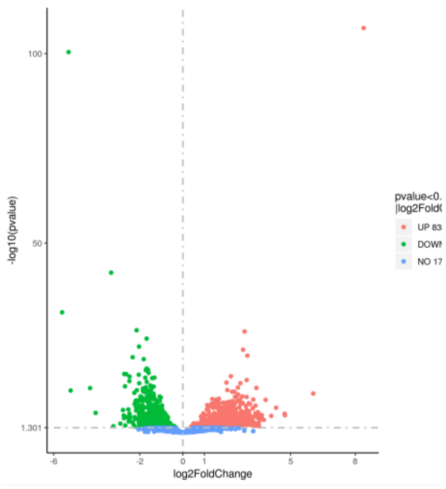

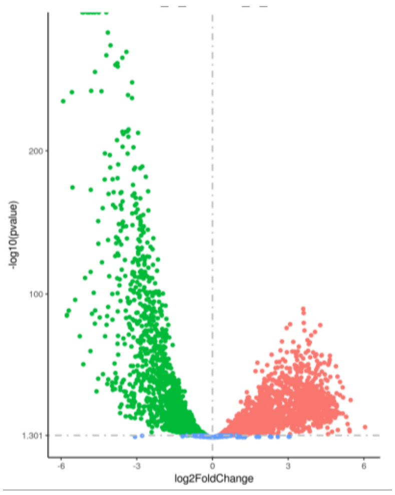

A **B**

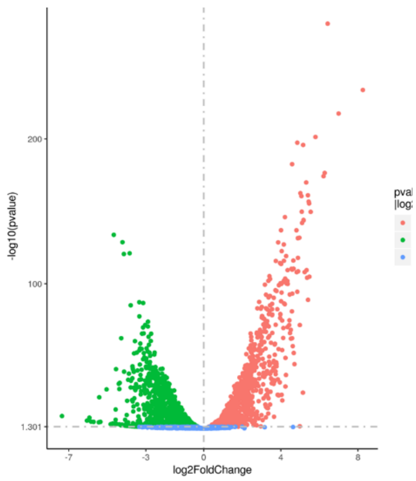

C

Figure S1: Volcano plots for (A) *L. monocytogenes* 10403S WT vs Δ*ctaP* in basal DM, (B) WT grown in basal DM vs DM with 1.57 mM L-cysteine, and (C) WT grown in basal DM vs 3.67 mM L-cysteine under anaerobic conditions

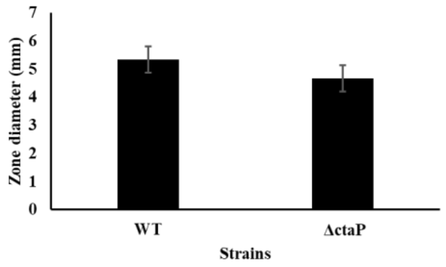

Figure S2: Phospholipase activity of *L. monocytogenes* 10403S WT and Δ*ctaP* on ALOA plates (Trafalgar Scientific, UK), incubated at 37 °C. Zones from 3 biological replicates were measured, and average diameters and standard deviations are presented.

Table S1: Summary of RNA-seq alignment.

Sample name	Raw reads	Clean reads	Raw Bases	Clean Bases	Error rate	Q20	Q30	GC content
WT in DM	16370812	16258662	2.46G	2.44G	0.02	98.18	94.39	39.42
WT in DM	13468558	13348956	2.03G	2.01G	0.02	98.11	94.26	39.43
WT in DM	16669806	16273724	2.51G	2.45G	0.02	98.27	94.63	39.63
ΔctaP in DM	17907818	17770786	2.69G	2.67G	0.02	98.24	94.54	39.05
ΔctaP in DM	17730138	17511014	2.66G	2.63G	0.02	98.3	94.71	39.93
ΔctaP in DM	19354470	19108730	2.91G	2.87G	0.02	98.21	94.49	39.39
WT in 1.57 mM L-cysteine	15588650	15333328	2.3G	2.3G	0.01	99.26	97.7	39.8
WT in 1.57 mM L-cysteine	17596022	17358002	2.64G	2.61G	0.03	97.7	93.18	39.81
WT in 1.57 mM L-cysteine	20843948	20685484	3.13G	3.11G	0.02	98.08	94.15	40.72
WT in 3.67mM L-cysteine	17905500	17492754	2.69G	2.63G	0.02	98.4	94.92	40.52
WT in 3.67mM L-cysteine	13278158	12972430	2.0G	1.95G	0.02	98.41	94.97	40.86
WT in 3.67mM L-cysteine	14885110	14769646	2.24G	2.22G	0.02	98.16	94.33	40.58

Q20: percentage of bases with a Phred value > 20; Q30: percentage of bases with a Phred value > 30

Table S2: Transcription of biofilm formation-related genes in WT grown in 1.57 mM L-cysteine-containing DM and 3.67 mM L-cysteine-containing DM compared

to non-supplemented DM, and in Δ*ctaP* compared to WT grown in DM under anaerobic conditions

**Gene symbol**	**Locus tag**	**Gene ID for 10403S**	**Log_2 fold-change Δ*ctaP* vs WT in DM**	**Log_2 fold-change WT in 1.57 mM vs DM**	**Log_2 fold-change WT in 3.67 mM vs DM**	**Gene description**
ltaP	*lmo0644*	LMRG_RS03235	+0.24	-2.02*	-0.47*	Lipoteichoic acid primase LtaP
asnB	*lmo1663*	LMRG_RS08290	-0.40	-1.24*	-1.25*	Asparagine synthase (glutamine-hydrolyzing)
galE	*lmo2477*	LMRG_RS12575	-1.50*	-0.80*	-0.76*	UDP-glucose 4-epimerase GalE
purD	*lmo1764*	LMRG_RS08910	-0.47	-0.47*	-0.15	Phosphoribosylamine--glycine ligase
purF	*lmo1768*	LMRG_RS08930	-0.21	+1.11*	0.00	Amidophosphoribosyltransferase
purH	*lmo1765*	LMRG_RS08915	-0.18	+0.68*	+0.19	Bifunctional phosphoribosylaminoimidazolecarboxamide formyltransferase/IMP cyclohydrolase
purL	*lmo1769*	LMRG_RS08935	+0.35	+2.18*	-0.15	Phosphoribosylformylglycinamidine synthase subunit PurL
purN	*lmo1766*	LMRG_RS08920	-0.04	+1.17*	-0.29	Phosphoribosylglycinamide formyltransferase
icd	*lmo1566*	LMRG_RS07795	-0.97*	-1.59*	+0.39*	NADP-dependent isocitrate dehydrogenase
	lmo1373	LMRG_RS06830	+0.29	+0.12	+0.87*	Alpha-ketoacid dehydrogenase subunit beta
	lmo1885	LMRG_RS09525	-1.20	-2.28*	-1.30*	Xanthine phosphoribosyltransferase
acnA	*lmo1641*	LMRG_RS08180	-0.37	-0.63*	+3.09*	Aconitate hydratase AcnA
	lmo2547	Novel00419	+0.44	+0.83*	-2.22*	Homoserine dehydrogenase

pgdA	*lmo0415*	LMRG_RS02135	+0.19	-2.71*	1.02*	Peptidoglycan N-acetylglucosamine deacetylase PgdA
	lmo0028	LMRG_RS00140	+1.22*	+2.48*	-0.06	LD-carboxypeptidase
sipZ	*lmo1271*	LMRG_RS06320	-1.01	-1.47*	-1.82*	Type I signal peptidase SipZ
	lmo2500	LMRG_RS12690	-0.73*	-0.81*	-0.56*	ATP-binding protein
	lmo2100	LMRG_RS10610	+1.73*	+3.06*	-0.91	PLP-dependent aminotransferase family protein family
	lmo1662	LMRG_RS08285	+1.51	+1.26*	+0.03	Class I SAM-dependent methyltransferase
polA	*lmo1565*	LMRG_RS07790	-1.23*	-1.74*	+0.45*	DNA polymerase I
	lmo2361	LMRG_RS11900	-0.51	-0.50*	-0.11	Rrf2 family transcriptional regulator
atpD	*lmo2529*	LMRG_RS12835	-1.62*	-3.58*	-0.80*	F0F1 ATP synthase subunit beta
plsX	*lmo1809*	LMRG_RS09150	-0.93	-1.17*	-2.42*	Phosphate acyltransferase PlsX
Novel	*lmo2785*	Novel00467	+1.83*	+2.97*	+0.74	Catalase
	lmo0358	LMRG_RS01850	+2.92*	+4.40*	+1.41*	Fructose-specific PTS transporter subunit EIIC
	lmo0028	LMRG_RS00140	+1.22*	+2.48*	-0.06	LD-carboxypeptidase
esaA	*lmo0057*	LMRG_RS00290	+1.92*	+3.88*	-1.25*	Type VII secretion protein EsaA
	lmo2826	LMRG_RS14380	+2.56*	+3.71*	+1.68*	*lmo2826* family MFS transporter
	lmo2056	LMRG_RS10385	+1.80	-0.67*	+0.45*	CAP domain-containing protein
Novel		Novel00016	+0.56	+0.11	+4.21*	Biofilm formation stimulator VEG

	lmo0189	LMRG_RS00910	+0.63	-0.56	+4.15*	Veg family protein
sugR	*lmo0852*	LMRG_RS04265	+0.36	+1.55*	-1.32*	Efflux SMR transporter transcriptional repressor SugR
	lmo0024	LMRG_RS00120	+3.08*	+4.88*	-0.34	PTS system mannose/fructose/sorbose family transporter subunit IID
	lmo0374	LMRG_RS01935	+2.38*	+3.60*	+4.27*	PTS sugar transporter subunit IIB
	lmo0544	LMRG_RS02720	+3.66*	+5.48*	+5.15*	PTS glucitol/sorbitol transporter subunit IIC
	lmo2434	Novel00393	-0.43	+0.86*	-2.16*	Pyridoxal-dependent decarboxylase conserved domain
secY		LMRG_RS13315	-0.14	-1.93*	+0.55*	Preprotein translocase subunit SecY
	lmo2192	LMRG_RS11120	+0.04	-2.64*	-1.21*	ATP-binding cassette domain-containing protein
	lmo1211	LMRG_RS06005	+1.06	+2.67*	-2.39*	DMT family transporter

Asterisks indicate a significant difference (*padj* < 0.05).
"-" indicates downregulation while "+" indicates upregulation

Table S3: Transcription of flagella and chemotaxis genes in WT grown in 1.57 mM L-cysteine-containing DM and 3.67 mM L-cysteine-containing DM compared to non-supplemented DM, and in Δ*ctaP* compared to WT grown in DM under anaerobic conditions

**Gene symbol**	**Locus tag**	**Gene ID for 10403S**	**Log_2 fold-change WT in 1.57 mM vs DM**	**Log_2 fold-change WT in 3.67 mM vs DM**	**Log_2 fold-change Δ*ctaP* vs WT in DM**	**Gene description**
cheR	*lmo0683*	LMRG_RS03430	+3.45*	-0.93*	+1.57	Protein-glutamate O-methyltransferase CheR

fliN	*lmo0698*	LMRG_ RS0348 0	-2.96*	-1.70*	-1.54*	Flagellar motor switch protein FliN
fliM		LMRG_ RS0351 0	-2.94*	-1.88*	0.07	Flagellar motor switch protein FliM
fliG	*lmo0714*	LMRG_ RS0358 5	-1.88*	-2.56*	-1.47*	Flagellar motor switch protein FliG
flgE		LMRG_ RS0350 0	-3.07*	-1.85*	-1.45*	Flagellar hook protein FlgE
flgB	*Lmo0710*	LMRG_ RS0356 5	-3.47*	-2.73*	-1.50*	Flagellar basal body rod protein FlgB
flis	*lmo0708*	LMRG_ RS0355 5	-3.47*	-3.70*	-1.46*	Flagellar protein FliS
rpoD		LMRG_ RS0723 5	-3.38*	0.28	+1.19*	RNA polymerase sigma factor RpoD
flgL	lmo0706	LMRG_ RS0354 5	-3.16*	-2.90*	-1.30*	Flagellar hook-associated protein

Asterisks indicate a significant difference (*padj* < 0.05).
"-" indicates downregulation while "+" indicates upregulation

Table S4: Transcription of the genes related to cysteine transport and quorum sensing in Δ*ctaP* compared to WT in DM, in 1.57 mM L-cysteine-containing DM and 3.67 mM L-cysteine-containing DM compared to non-supplemented DM under anaerobic conditions

**Gene symbol**	**Locus tag**	**Gene ID for 10403 S**	**Log_2 fold-change Δ*ctaP* vs WT in DM**	**Log_2 fold-change Δ*ctaP* in 1.57 mM vs DM**	**Log_2 fold-change Δ*ctaP* in 3.67 mM vs DM**	**Gene description**
tcyK	*lmo2349*	LMRG_RS 11840	+2.55*	-0.61	-0.95	Amino acid ABC transporter substrate-binding protein
tcyL	*lmo2348*	LMRG_RS 11835	+1.54*	+0.47	+0.04	Amino acid ABC transporter permease
tcyM	*lmo2347*	LMRG_RS 11830	+1.46*	-0.44	-0.61	Amino acid ABC transporter permease
tcyN	*lmo2346*	LMRG_RS 11825	+1.60*	-0.17	-0.51	Amino acid ABC transporter ATP-binding protein

CymR	*lmo15 15*	LMR G_RS 07540	+0.58*	-1.02*	-0.65	Rrf2 family transcriptional regulator
oppA	*lmo01 52*	LMR G_RS 00730	+3.15	-3.34	-3.06	Peptide ABC transporter substrate-binding protein PF00496:Bacterial extracellular solute-binding proteins
oppB	*lmo21 95*	LMR G_RS 11135	-0.74*	-1.28*	-0.70	ABC transporter permease
oppC		LMR G_RS 11130	-0.45	-1.19*	-0.73	ABC transporter permease
oppD		LMR G_RS 11125	-0.08	-1.84*	-1.24	ABC transporter ATP-binding protein
oppF	*lmo21 92*	LMR G_RS 11120	+0.04	-2.08*	-1.41	ATP-binding cassette domain-containing protein
luxS	*lmo12 88*	LMR G_RS 06405	+0.08	-2.63*	-2.41*	S-ribosylhomocysteine lyase

*Asterisks indicate a significant difference (*padj* < 0.05).
"-" indicates downregulation while "+" indicates upregulation

Table S5: Transcription of *dltA*, *dltB, cheA* and *cheY* in Δ*ctaP* compared to WT in DM, in 1.57 mM L-cysteine-containing DM and 3.67 mM L-cysteine-containing DM compared to non-supplemented DM under anaerobic conditions

**Gene symbol**	**Locus tag**	**Gene ID for 10403S**	**Log_2 fold-change Δ*ctaP* vs WT in DM**	**Log_2 fold-change Δ*ctaP* in 1.57 mM vs DM**	**Log_2 fold-change Δ*ctaP* in 3.67 mM vs DM**	**Gene description**
cheA	*lmo0692*	LMRG_RS0 3475	-1.51*	-0.44	-0.54	Chemotaxis protein CheY
cheY	*lmo0691*	LMRG_RS0 3470	-1.35*	-0.74	-0.23	Chemotaxis protein CheA
dltA		LMRG_RS04 905	-1.50 *	-2.19*	+0.62	D-alanine--poly (phosphoribitol) ligase subunit DltA
dltB	*lmo0973*	LMRG_RS04 900	-1.78 *	-2.30*	-0.03	D-alanyl-lipoteichoic acid biosynthesis protein DltB
